# Behavioral changes in thoroughbred horses following pre-training: insights from novel object tests and activity monitoring under field conditions

**DOI:** 10.3389/fvets.2026.1927282

**Published:** 2026-09-21

**Authors:** Lara Pinetzki, Jule Menze, Fabienne Eichler, Isabel Brüsewitz, Tina Engler, Saskia Stutzke, Roswitha Merle, Gundula Hoffmann, Mechthild Wiegard, Christa Thöne-Reineke, Katharina Kirsch

**Affiliations:** 1 Institute of Animal Welfare, Animal Behavior and Laboratory Animal Science, Freie Universität Berlin, Berlin, Germany; 2 Department Sensors and Modeling, Leibniz Institute for Agricultural Engineering and Bioeconomy (ATB), Potsdam, Germany; 3 Institute of Movement and Training Science in Sports II, Leipzig University, Leipzig, Germany; 4 Institute of Veterinary Epidemiology and Biostatistics, Freie Universität Berlin, Berlin, Germany

**Keywords:** animal welfare, behavioral test, equine, racehorse, training

## Abstract

Racehorse training starts with pre-training, which is conducted at a very early age and remains controversial from an animal welfare perspective. This study investigated how a 12-week pre-training program influenced the behavior of young Thoroughbred horses. Horses were assessed before (A) and after (B) pre-training, which progressed from habituation and handling to ridden exercise and canter work on a racetrack. Behavioral responses were evaluated using standardized novel object tests and continuous 24-h stall monitoring via inertial measurement unit sensors. Novel object test behavior was video-recorded and analyzed using a validated ethogram comprising 34 behavioral parameters. Following pre-training, horses showed significantly increased locomotor activity during novel object tests, including longer durations of trot (A: 16.1 ± 6.7 s; B: 41.6 ± 6.7 s; *p* < 0.01), gallop (A: 1.9 ± 1.6 s; B: 11.4 ± 1.6 s; *p* < 0.001), and bucking (A: 1.0 ± 2.8 s; B: 22.2 ± 2.8 s; *p* < 0.001). Tactile exploration also increased, reflected in more frequent muzzle contact with the object (A: 2.2 ± 0.8; B: 6.0 ± 2.0; *p* < 0.05), despite a reduction in the total number of approaches (A: 8.1 ± 0.9; B: 6.3 ± 0.7; *p* < 0.05), suggesting altered patterns of interaction with the novel object following pre-training. Stall behavior correlated with responses in the novel object test, supporting the consistency of individual behavioral tendencies across contexts. Principal component analysis revealed four main profiles: highly active/offensive behavior, exploratory engagement, vigilance/defensive behavior, and calm observation. Notably, inter-individual variability between individuals increased after pre-training. The shift toward increased locomotion and tactile exploration suggests adaptive behavioral modulation in response to early training. While some horses transitioned toward more active and confident profiles, others remained stable, indicating that certain traits may be inherent whereas others are experience-dependent. These findings highlight the importance of multidimensional behavioral assessment and demonstrate that integrating experimental and continuous monitoring approaches can improve the interpretation of novel object test responses and support evidence-based equine welfare assessment.

## 1 Introduction

Public concern regarding equine wellbeing has experienced a notable rise, leading to increased attention to animal welfare issues affecting horses. Consequently, extensive public debate has emerged about the use of horses, with the racehorse industry in particular facing repeated criticism. One major point of concern is the early initiation of training and racing in thoroughbred horses, which commonly begins at the age of two (1). There is a common debate in the industry about whether starting race training at 2 years of age is beneficial or detrimental to the animal's career and growth (2). Thoroughbreds in flat racing typically begin training at 1.5 to 2 years of age, whereas national hunt or jump racing—prevalent in Great Britain—commences at 3 to 4 years (3). According to the results of a recent survey, thoroughbred horses in Germany typically enter pre-training at the age of 18 months (4). Pre-training programs are intended to facilitate young horses' transition into their future roles as racehorses by gradually and professionally acclimatizing them to human handling and race-specific equipment. This raises the question of whether pre-training influences equine behavior and whether such effects can be measured using a novel object test. Visser et al. (5) tested untrained and trained horses using the novel object test and found that the untrained horses reacted more pronounced to an unknown object.

An essential component of evaluating equine wellbeing is the systematic observation of behavioral patterns. Welfare assessments are most effective when multifaceted, as single measures cannot sufficiently capture an animal's overall welfare status (3). Among the available behavioral assesments, the novel object test has been a widely used method for investigating behavioral reactivity and fearfulness in equine research. However, despite its widespread application, methodological differences between studies complicate the interpretation and comparison of findings. Previous research has demonstrated that horses' responses in novel object tests are influenced by external factors. For example, the testing environment has been shown to significantly affect behavioral responses, emphasizing the importance of standardized testing conditions when comparing individual horses (6). Likewise, the characteristics of the novel object may influence behavioral responses. According to Hall and Cassaday (7) certain colors have the potential to induce an increased adverse reaction in equines, namely yellow, white, black, and blue. Others studies have reported no differences between objects of different colors but similar shapes (8, 9). Similarly, previous work has shown that horses do not necessarily generalize between novel objects that differ in both color and shape (8). Collectively, these findings indicate that the influence of object characteristics on behavioral responses remains insufficiently understood and that methodological variation may contribute to inconsistent results across studies.

Furthermore, most previous studies have focused exclusively on horses‘ responses during the novel object test itself, whereas relatively little is known about how these responses relate to behavior expressed in the home environment. Determining whether behavioral patterns observed during standardized behavioral testing are reflected in everyday stable behavior would improve the interpretation of novel object tests as indicators of individual behavioral tendencies and welfare. Therefore, the present study investigated horses' responses to different novel objects under standardized conditions and examined their relationship with behavioral patterns expressed in the stable.

In addition to behavioral testing, continuous behavior monitoring provides valuable insights into equine welfare. While camera systems might be limited to recording within the stall and require optimal lighting conditions, activity sensors offer distinct advantages. These sensors are capable of recording continuously over a 24-h period, allowing a comprehensive assessment of horses' behavior both inside and outside the box. Time budgets play a decisive role in assessing animal welfare factors (3). Changes in daily time budgets can reflect external stressors, unsuitable housing conditions or reduced wellbeing, making their analysis a valuable tool for detecting early signs of welfare impairment in horses (10–12).

This study aims to evaluate the influence of pre-training on young racehorses' behavior using a combination of the novel object test and behavior monitoring over 24 h. It is hypothesized that pre-training influences the behavior of young racehorses, specifically leading to a reduction in behaviors associated with stress and anxiety. Furthermore, object characteristics, namely color and shape, are expected to affect the extent of direct interaction during the novel object test, whereas no significant effect of sex on object-related behavioral responses is anticipated. In addition, pre-training is expected to result in measurable changes in the stall-based time budget, while fundamental behaviors such as feeding and resting are assumed to remain stable irrespective of sex.

## 2 Material and methods

Ethical permission was approved by the competent authority of the federal state in which the study was conducted, under the reference number 2.4.2.2.-15-2023.

### 2.1 Animals

The study took place on a stud farm for thoroughbred horses and included 16 mares and 14 stallions (*n* = 30), aged between 18 and 24 months. The horses were housed in individual stalls equipped with windows and were permitted to access pastures in small groups on a regular basis. The single boxes enabled continuous visual, olfactory, and auditory interaction. Minor physical contact was possible through the steel bars between individual stalls. The horses were fed hay three times per day and a proprietary high-energy feed mixture formulated for racehorses three times daily. Following their arrival at the pre-training stable, the horses were given an acclimation period to adapt to the new environment and management procedures. At the beginning of the study, all horses were habituated to daily interactions with halters, ropes and bridles, but had not been further prepared for riding. After several days of habituation, they completed their first Novel Object Test.

### 2.2 Pre-training

The stud farm specialized in the pre-training of racehorses. Habituation of yearlings began between autumn and spring with introductions to human proximity and riding equipment. The pre-training period typically spans 12–16 weeks. It first involves the habituation of the horse to saddle and tack followed by the mounting of a rider and guided walking, and subsequent walking and trotting in the horse walker. After the first few weeks of habituation, the horses are initially ridden in an indoor arena until they are confident in all three gaits to progress into regular cantering sessions on a 1,400 m outdoor race track.

Pre-training may also include individual rest days for the horses. It is imperative to acknowledge that thoroughbred racehorses are not trained in isolation; rather, horses are always trained in strings of horses known as “lots”.

### 2.3 Novel object test

Two novel object tests were performed, the first (A) prior to the beginning of the habituation and the second (B) after a 4-month pre-training period. The behavioral tests were conducted in a covered round pen inside a horse walker. The round pen measured 14 m in diameter and was filled with a sand mixture. Its outer boundary consisted of a 2-m-high wooden wall. For the first test, the same pool raft shaped like a “mermaid's tail” (approx. 170 × 85 × 100 cm) was used as an unknown object (6). The raft was a light blue inflatable ring featuring a fish tail with pink-colored tips.

For the second novel object test, three different, previously unseen objects were used. All horses had been exposed to the same novel object during the first test; therefore, new objects were introduced in the second test to prevent habituation and to examine the effects of object color and shape on equine behavior. The object “blue” (approx. 170 × 85 × 50 cm) was a modified version of the “mermaid's tail,” with the fish tail rotated inside the swimming ring. The pool raft “fish” was orange-white in color and measured approx 155 × 86 × 39 cm. The third object, “lama” (approx. 165 × 65 × 100 cm), was a blue inflatable ring with a raised head. In the second test, the three objects were assigned randomly. A total of 25 horses participated: six horses encountered the “blue” object, 10 the “fish,” and nine the “lama.” A second test could not be conducted with five horses as two horses had left the stud farm, and three were unable to participate due to illness.

The experimental protocol was identical for all subjects. The equines have not been specifically habituated to the test arena, but they have been to the horse walker, which circumvents the round pen. Each horse was collected at the stable and guided into the round pen by a familiar handler. At the center of the arena, there was already a novel object positioned. After entering, the horse was positioned facing the exit and then released from the lead rope. The handler immediately left the testing area, the door was closed, and the horse remained alone with the object for 10 min. After the 10-min test period, the horse was retrieved and returned to its stall. Before the next horse entered, any horse manure was collected and the surface was leveled to remove any irregularities.

#### 2.3.1 Assessment of behavior during the novel object test

Behavior was recorded using two GoPro Hero 11 Black action cameras (GoPro, Inc., San Mateo, CA, USA), mounted opposite each other on the outer edge of the horse walker. All videos were randomized and blinded to be assessed by an independent observer. A previously defined standard ethogram (Table 1), which was validated in a previous study, was applied for the evaluation with 11 behaviors coded relating to interactions with the novel object, and 23 behaviors unrelated to the novel object (6). The software “The Observer XT 16” (Noldus Information Technology, Wageningen, Netherlands, version 16.0.1203) was used to code the type, number and duration of all behaviors recorded on video (Table 1). The behavioral repertoire of the horses was systematically documented, with each behavior defined in precise terms. The initial fixation, approach, and contact of the horse with the unfamiliar object were documented, along with the manner in which the horse interacted with the novel object. In addition to the video-based data collection, activity sensors (GT9XLink, ActiGraph, Pensacola, FL, USA) recorded relevant activities during the novel object test such as standing, walking, trotting, cantering and rolling using a deep neural network, as described by Kirsch et al. (10).

**Table 1:** Ethogram for the observed behaviors during the novel object test.

Observed behavior	Parameter	Abbreviation	Description
Latency			
Latency time until the first fixation of the novel object	duration (s)	latency first fix obj	Horse is standing or moving, ears toward object, staring at object, object is in horse's binocular field of vision
Latency time until the first approach to the novel object	duration (s)	latency first approach obj	The object is in the horse's binocular field of vision, the horse looks at the object and takes one step toward the object (gait does not matter)
Latency time until the novel object is touched for the first time	duration (s)	latency first touch obj	A part of the horse's body touches the object intentionally (accidental tail flicking and touching the object does not count)
Interaction with the novel object			
Fixating the novel object	duration (s)	duration fix obj	Horse is standing or moving, ears toward object, staring at object, object is in horse's binocular field of vision, as soon as the horse changes the direction of gaze and then fixates the object again, this is counted as separate fixating events
	number (count)	nr fix obj	
Approaching the novel object	number (count)	nr approach obj	The object is in the horse's binocular field of vision, the horse looks at the object and takes one step toward the object (gait does not matter), each first step after a standstill of >2 s is counted as separate approaching events (subsequent steps without a standstill in between are counted as one event)
Sniffing on the novel object	duration (s)	duration sniff obj	Muzzle 20 cm away from the novel object, if there is no clear indication of the muzzle touching the object or simply sniffing the object, this behavior should be coded accordingly, as soon as the muzzle of the horse moves >20 cm away from the object and then sniffs on the object again, this is counted as separate sniffing events
	number (count)	nr sniff obj	
Touching the novel object with the muzzle	number (count)	nr touch obj muzzle	Touching with the muzzle, which leads to a visible movement of the object, as soon as the horses muzzle no longer touches the object for >2 s and then touches the object again this is counted as separate touching events
Lifting the novel object	number (count)	nr lift obj	Lifting the novel object from the ground using the muzzle
Touching the novel object with the forelimb	number (count)	nr touch obj forelimb	Pawing or kicking with one front leg toward the object
Kicking the novel object with the hindlimb	number (count)	nr kick obj	Kicking with hind leg toward the object, without necessarily touching it
Behaviors unrelated to the novel object			
Sniffing the ground	duration (s)	duration sniff ground	Horse is standing or moving with lowered head and sniffing on the ground (nose below the shoulder)
Chewing/licking	number (count)	nr chewing	Movement with muzzle is visible
Grinning/flehming	number (count)	nr flehming	Lifting the head up/forward with curling of the upper lip, looks like horse is smiling, incisors visible
Neighing/whinnying	number (count)	nr neighing	Loud sound, most times while head is up, whole body is shaking
Squeaking	number (count)	nr squeaking	High and loud sound
Blowing	number (count)	nr blowing	Noisy exhalation, nostrils dilated, audibly increased, intermittent exhalation, forceful snorting
Snorting	number (count)	nr snorting	Noisy exhalation, fluttering nostrils
Head tossing	number (count)	nr head tossing	Shaking the head up and down, while standing or walking, aimed horizontal or vertical movement of the head
Tailswishing	number (count)	nr tailswishing	Active moving tail suddenly from side to side, during standing or walking
Pawing	number (count)	nr pawing	Arching action of horse's foreleg which strikes the ground
Stomping	number (count)	nr stomping	Suddenly flexing and then extending any limb to the ground
Lying down without rolling	number (count)	nr lying down	Abdomen touches floor and all extremities flexed
Lying down with rolling	number (count)	nr rolling	Rolling on the ground, starts when abdomen touchs the ground
Standing at the exit	duration (s)	duration standing exit	Standing in the entrance/exit area, entire head is inside the imaginary triangle between object and door, as soon as the horse leaves the entrance/exit area, then moves back in this area and stands for >2 s, this is counted as separate events
	number (count)	nr standing exit	
Defecating	number (count)	nr defecating	
Urinating	number (count)	nr urinating	
Rearing	number (count)	nr rearing	The horse stands up on its hind legs with the forelegs off the ground
Bucking	duration (s)	duration bucking	No defined gait, no clear rhythm recognizable, the horse can kick, jump, lowered head neck position, tail flap, head flap, round back, 2 or 4 legs off the ground at the same time
	number (count)	nr bucking	
Yawning	number (count)	nr yawning	Open the mouth wide, head often back, eye closed/eye rolling for a few second
Flinching	number (count)	nr flinching	Sudden reflexive contraction of muscles, involuntary jerking movement with or without a single jump away from the object (more than one jump is bucking)
Shaking	number (count)	nr shaking	e.g., after rolling
Rolling^*^	duration (s)	duration ROL	Rolling on the ground
Walking^*^	duration (s)	duration WAL	
Trotting^*^	duration (s)	duration TRO	
Galloping^*^	duration (s)	duration GAL	
Active^*^	duration (s)	duration_ACT	Trot and gallop
Standing alert^*^	duration (s)	duration SAL	Standing in a fixed and rigid position, head raised (poll above the withers), very little movement, attentively observing an object or sound

^*^activities analyzed based on activity sensor data.

#### 2.3.2 Sensor-based behavior assessment

In addition to the collection of video-based data, the horses were fitted with activity sensors (Figure 1) including an inertial measurement unit (IMU) with a 3-axis accelerometer and a gyroscope (GT9XLink, ActiGraph, Pensacola, FL, USA). The sensors were used for the purpose of data collection on the novel object test examination days, continuously recorded the horses' movements and positions over a 24-h period. The sensors were attached to the left side of the horse's neck using a collar and recorded relevant activities, including standing, walking, eating and lying in lateral and sternal recumbency, as exemplified by Kirsch et al. (10). The activities are presented as the proportion of time spent on a respective behavior per hour (/h). All horses were habituated to wearing the sensor collars for several hours prior. No instances of aversive behavior related to the attachment of the collars were observed.

**Figure 1:**
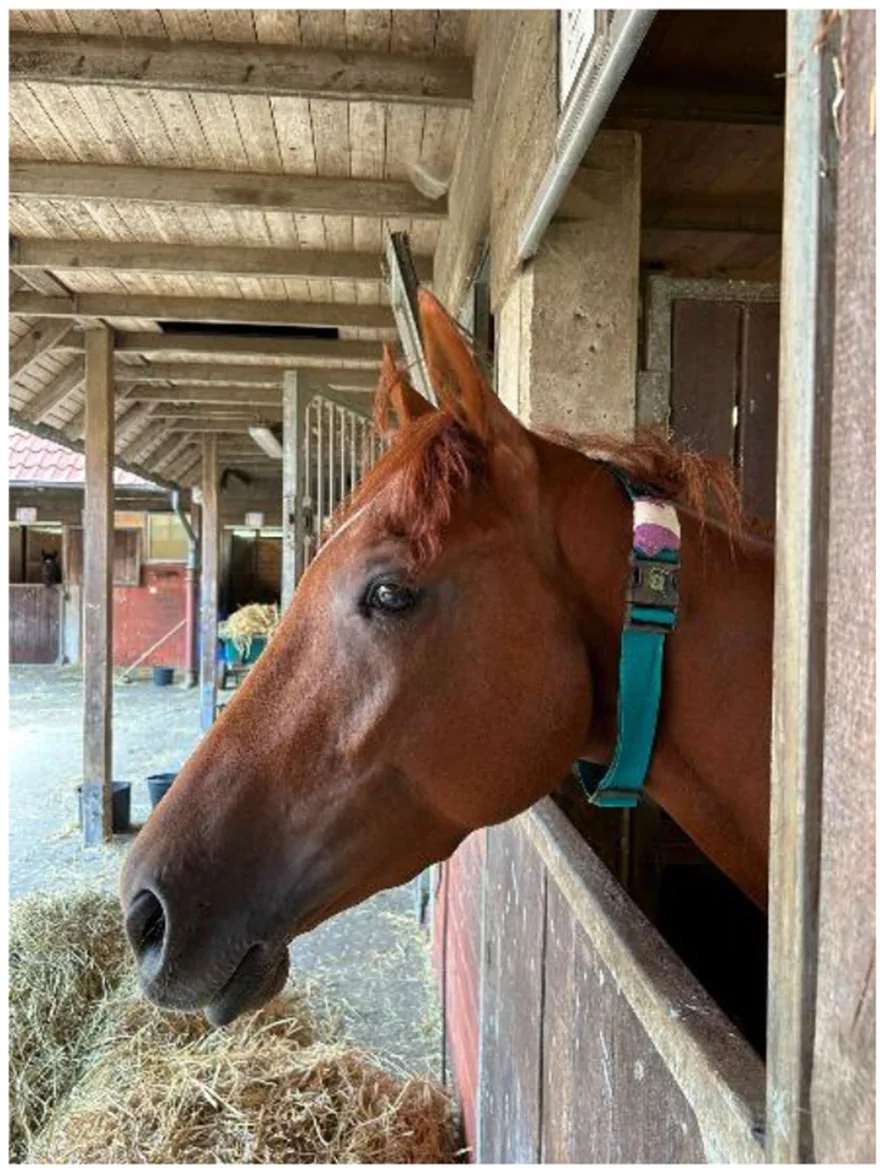
Activity sensor on the neck that measures acceleration along three axes.

### 2.4 Statistical analysis

The statistical analyses were conducted exclusively on the 25 horses that completed both novel object tests. Five horses were therefore excluded because they did not participate in the second test, as two had left the stud farm and three were unable to participate due to illness. Statistical analysis of the ethogram and the sensor data was performed using *R* statistical software (v4.4.0; *R* Core Team, 2024). Depending on the dependent variable being continuous (durations from the ethogram and activities per hour from the sensor data) or discrete (count data from the ethogram), linear or negative binomial generalized linear mixed effects models were fitted using the “lmer” or “glmer.nb” function. In order to evaluate the difference between the first test during the first weeks of habituation (Test A) and the second test at the end of the pre-training phase (Test B), the time point was included as a fixed effect. Further, to evaluate whether sex and object have a confounding effect on the behavior during the novel object test, sex, object as well as the interaction between test and sex were included as fixed effects. To account for multiple measurements in the same horses, the horse was included as a random effect. The significance of the fixed effects on the behavior during the novel object test was evaluated by comparing full models to models without the fixed effects by means of likelihood ratio tests using the “anova” function in *R*. In case of significance, *post hoc* pairwise comparisons were conducted using the, emmeans' package. For comparisons involving multiple factors, *p*-values were adjusted for multiple testing using Tukey's method. Normality of the error distributions of the linear regression models was evaluated by means of normal probability plots of the residuals. The results are presented as box plots and estimated marginal means. The results that are presented in Table 2 were provided as estimated marginal means ± standard error. In order to describe the behavior of the horses during the novel object tests as comprehensively as possible, a relatively large number of different behavioral parameters were recorded during this study. To reduce the dimensionality of the data and to find associations between different behavioral parameters, principal component analysis (PCA) of the ethogram and sensor data was conducted using the “prcomp” function in *R*. Subsequently, a kmeans cluster analysis of the coordinates of the different variables on the principal components (retaining only those components with an eigenvalue >1) was carried out. The resulting clusters were visualized in the loadings plot of the first two principal components (PC1 and PC2).

**Table 2:** Results of the novel object tests and stall behavior time budgets.

Observed behavior	Test A	Test B
Latency		
latency first fix obj (s)	7.3 ± 2.2^**^	26.0 ± 9.2^**^
latency first approach obj (s)	69.6 ± 29.5	85.2 ± 27.6
latency first touch obj (s)	432.6 ± 67.5	329.7 ± 51.5
Interaction with the novel object		
duration fix obj (s)	38.3 ± 3.9^***^	11.4 ± 3.9^***^
nr fix obj (count)	12.5 ± 1.4^***^	4.9 ± 0.7^***^
nr approach obj (count)	8.1 ± 0.9^*^	6.3 ± 0.7^*^
duration sniff obj (s)	6.1 ± 1.8	6.5 ± 1.8
nr sniff obj (count)	1.4 ± 0.3	2.3 ± 0.5
nr touch obj muzzle (count)	2.2 ± 0.8^*^	6.0 ± 2.0^*^
nr kick obj (count)	3.3e-5 ± 5.1e-5^***^	2.0 ± 0.6^***^
Behaviors unrelated to the novel object		
duration sniff ground (s)	142.0 ± 14.9	125.8 ± 14.9
nr sniff ground (count)	28.7 ± 2.2^**^	21.2 ± 1.7^**^
nr chewing (count)	0.6 ± 0.2	1.0 ± 0.3
nr neighing (count)	7.9 ± 1.7	9.1 ± 2.0
nr squeaking (count)	0.01 ± 0.01^***^	0.8 ± 0.4^***^
nr blowing (count)	28.3 ± 7.2	29.5 ± 7.5
nr snorting (count)	29.1 ± 7.0	30.4 ± 7.3
nr head tossing (count)	0.8 ± 0.2^***^	5.0 ± 1.1^***^
nr tailswishing (count)	6.1 ± 3.0	1.6 ± 0.8
nr pawing (count)	8.7 ± 3.6	17.1 ± 7.0
duration standing exit (s)	104.6 ± 13.3	124.4 ± 13.3
nr standing exit (count)	9.2 ± 1.0	10.3 ± 1.0
nr defecating (count)	2.0 ± 0.4^***^	1.1 ± 0.2^***^
duration bucking (s)	1.0 ± 2.8^***^	22.2 ± 2.8^***^
nr bucking (count)	0.3 ± 0.1^***^	4.4 ± 0.9^***^
nr flinching (count)	0.8 ± 0.2	0.5 ± 0.2
nr shaking (count)	0.2 ± 0.1	0.1 ± 0.1
duration WAL (s)	139.0 ± 9.9^***^	85.4 ± 9.9^***^
duration TRO (s)	16.1 ± 6.7^**^	41.6 ± 6.7^**^
duration GAL (s)	1.9 ± 1.6^***^	11.4 ± 1.6^***^
duration SAL (s)	110.2 ± 10.8	87.6 ± 10.8
Behavioral time budgets		
box EGR	0.408 ± 0.010	0.426 ± 0.010
box ETR	0.038 ± 0.004^*^	0.045 ± 0.004^*^
box WAL	0.021 ± 0.001^***^	0.013 ± 0.001^***^
box SAL	0.076 ± 0.005^***^	0.042 ± 0.005^***^
box REC	0.243 ± 0.012^**^	0.278 ± 0.012^**^

Asterisks indicate different levels of significance: ^*^p < 0.05, ^**^p < 0.01, ^***^p < 0.001.

## 3 Results

### 3.1 Behavior analysis during the novel object test

#### 3.1.1 Ethogram

The statistical analysis was conducted exclusively on the 25 horses that performed both novel object tests (A and B). The most meaningful finding was that the horses exhibited higher levels of activity in the second novel object test. Compared with the first test, horses showed significantly more trotting (Figure 2A) and galloping (Figure 2B), whereas walking was more prevalent during the first novel object test (Figure 2C). The horses also spent more time in the “standing alert” position during Test A (Figure 2D). In addition, horses displayed more squeaking (Figure 3A) and head tossing (Figure 3B), together with an increase in bucking behavior (Figures 3C, D), indicating an overall higher level of activity during the second novel object test.

**Figure 2:**
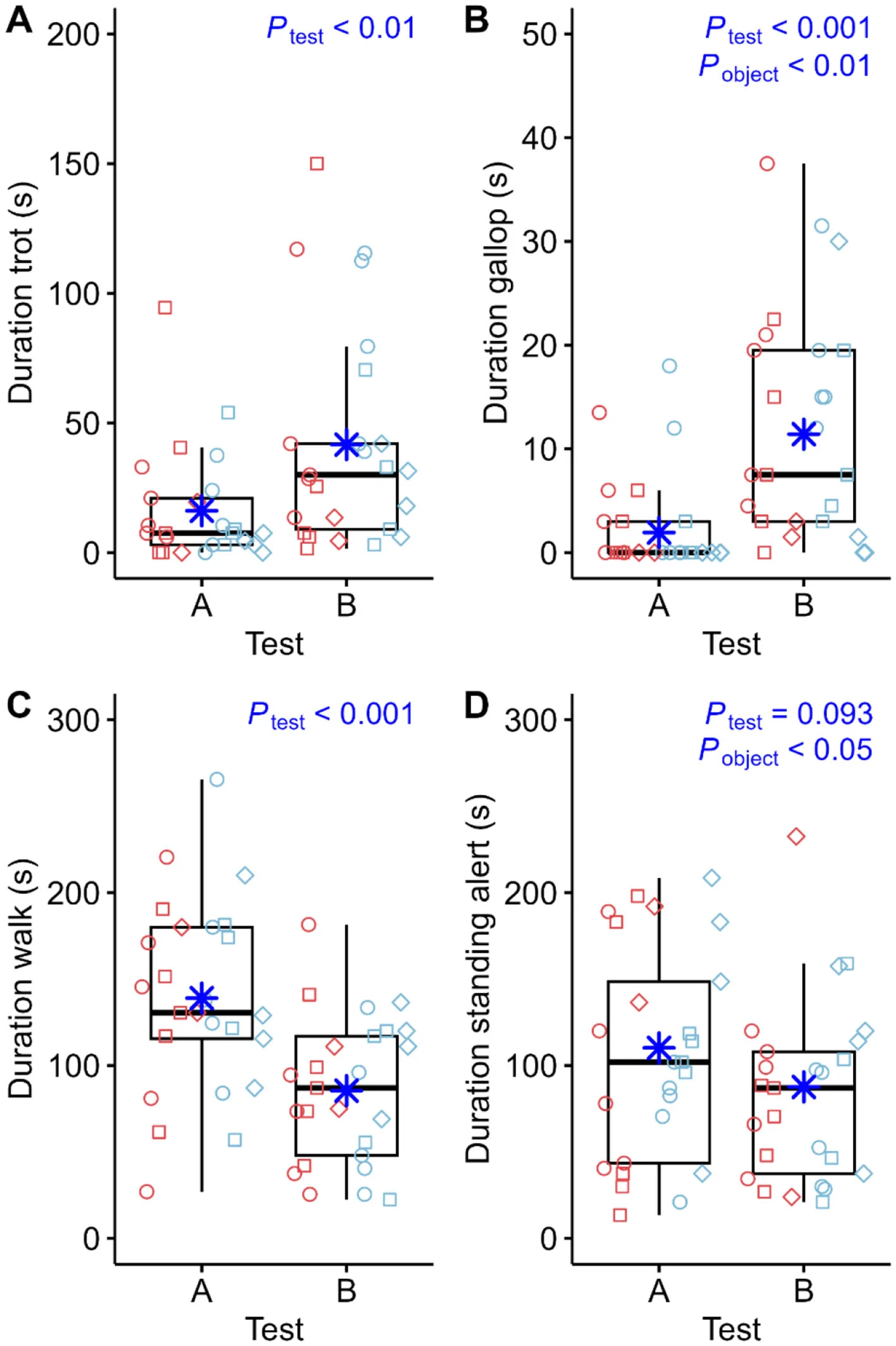
**(A)** Duration of trotting, **(B)** duration of galloping, **(C)** duration of walking, and **(D)** duration of standing alert compared between Test A (first novel object test) and Test B (second novel object test; *n* = 25). The box plots display the distribution of data by indicating the median and the interquartile range (IQR). Individual data points from mares (*n* = 12) are either shown as red circles (novel object test with object “fish”), red squares (novel object test with object “lama”) or red trapezoid (novel object test with object “blue”). Individual data points from stallions (*n* = 13) are shown in blue circles, squares and trapezoids accordingly. The marginal means estimated by the mixed effects models are shown as blue asterisks. *P*-values result from negative-binomial regression models.

**Figure 3:**
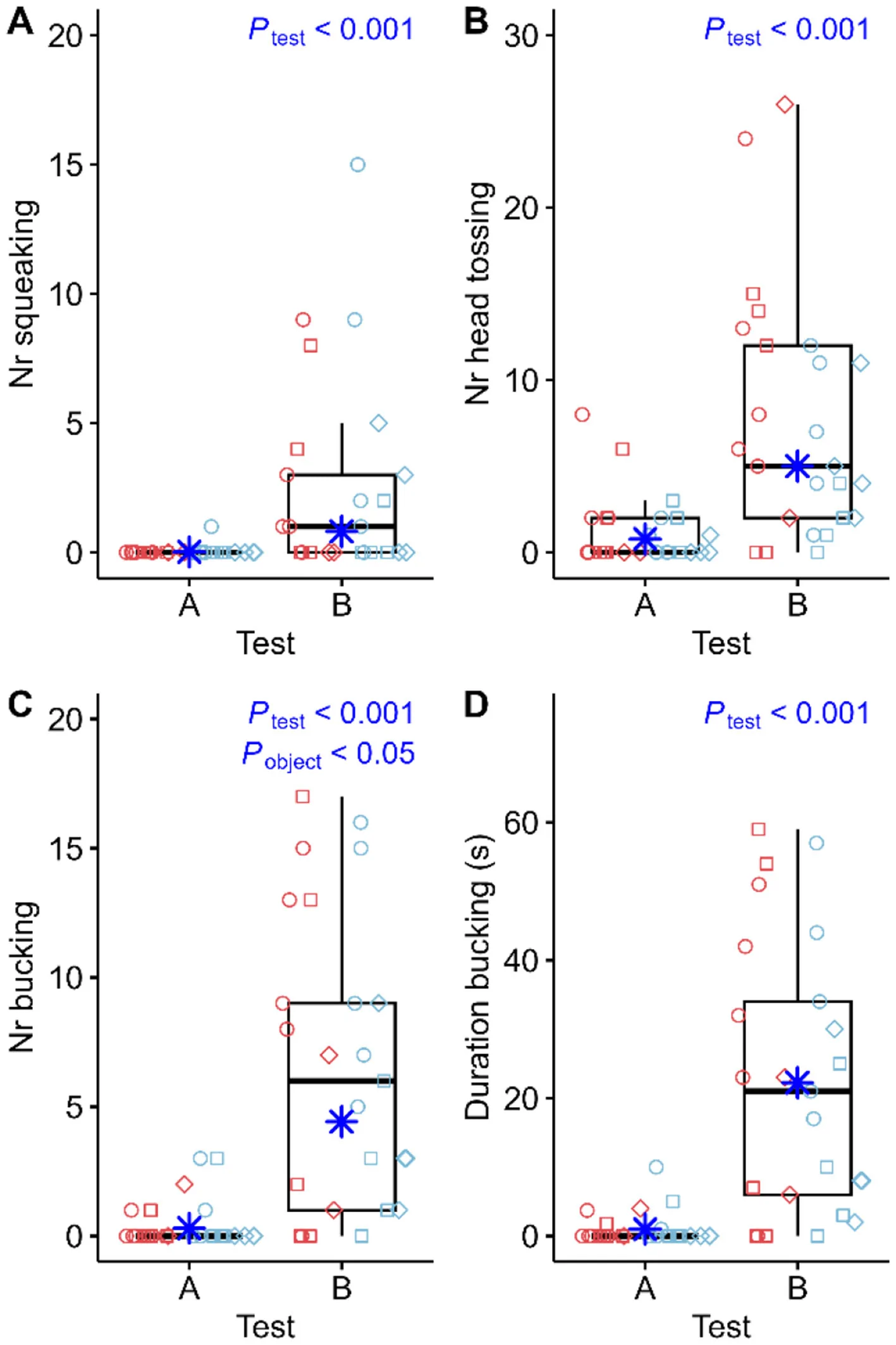
**(A)** Number of squeaking, **(B)** number of head tossing, **(C)** number of bucking, and **(D)** total duration of bucking compared between Test A (first novel object test) and Test B (second novel object test) (*n* = 25). The box plots display the distribution of data by indicating the median and the interquartile range (IQR). Individual data points from mares (*n* = 12) are either shown as red circles (novel object test with object “fish”), red squares (novel object test with object “lama”) or red trapezoids (novel object test with object “blue”). Individual data points from stallions (*n* = 13) are shown in blue circles, squares and trapezoid accordingly. The marginal means estimated by the mixed effects models are shown as blue asterisks. *P*-values result from negative-binomial regression models.

The latency to first fixation was longer in the second novel object test (Figure 4A). In addition, horses showed fewer fixations of the novel object (Figure 4B), a shorter total fixation duration (Figure 4C), and fewer approaches toward the object (Figure 4D). In contrast, the latency toward first approaches and first touches of the novel object did not differ between tests A and B. Likewise, no differences were found in lifting the novel object, touching it with a forelimb, or the overall occurrence of fixation and approach behaviors.

**Figure 4:**
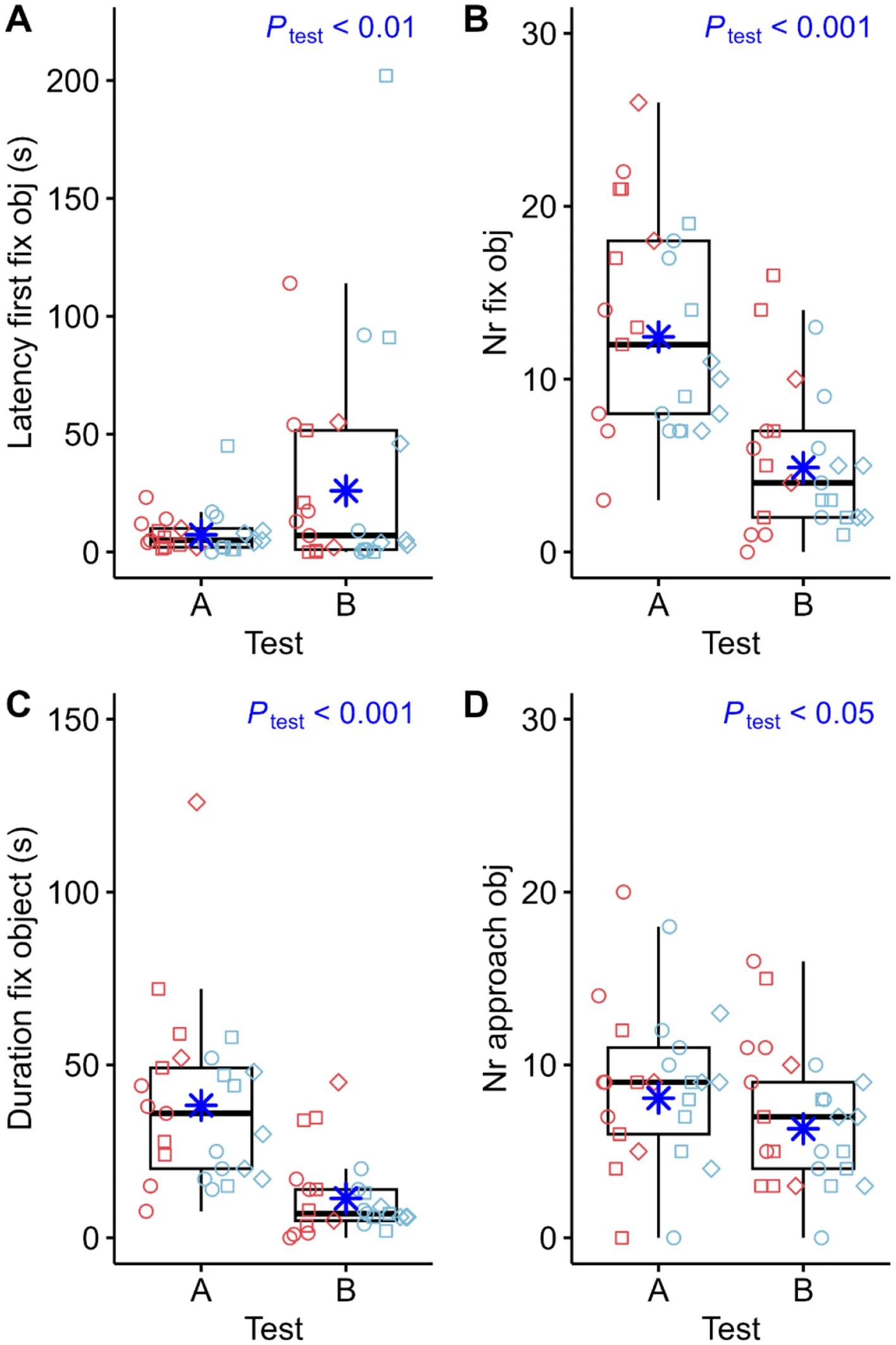
**(A)** Latency time until the first fixation of the object, **(B)** number of fixations of the object, **(C)** duration of fixation of the object, and **(D)** number of approaching the object compared between Test A (first novel object test) and Test B (second novel object test) (*n* = 25). The box plots display the distribution of data by indicating the median and the interquartile range (IQR). Individual data points from mares (*n* = 12) are either shown as red circles (novel object test with object “fish”), red squares (novel object test with object “lama”) or red trapezoid (novel object test with object “blue”). Individual data points from stallions (*n* = 13) are shown in blue circles, squares and trapezoids accordingly. The marginal means estimated by the mixed effects models are shown as blue asterisks. *P*-values result from negative-binomial regression models.

In the initial novel object test, 12 out of 25 horses, representing 48% of the total sample, touched the object within the allotted examination time. In the second novel object test, this number increased to 68% of the horses touching the object. Additionally, the horses touched the novel object more often with the muzzle in test B (Figure 5A). Kicking the object, which was observed only in one horse during the first novel object test, was shown more often in the second test (Figure 5B). A tendency toward more frequent sniffing of the object (Figure 5C) was also observed in the second novel object test, with a significant interaction between sex and test, and pairwise comparisons indicating that this difference was only observed in mares. Contrary, sniffing on the ground and defecating were observed less often in the second novel object test.

**Figure 5:**
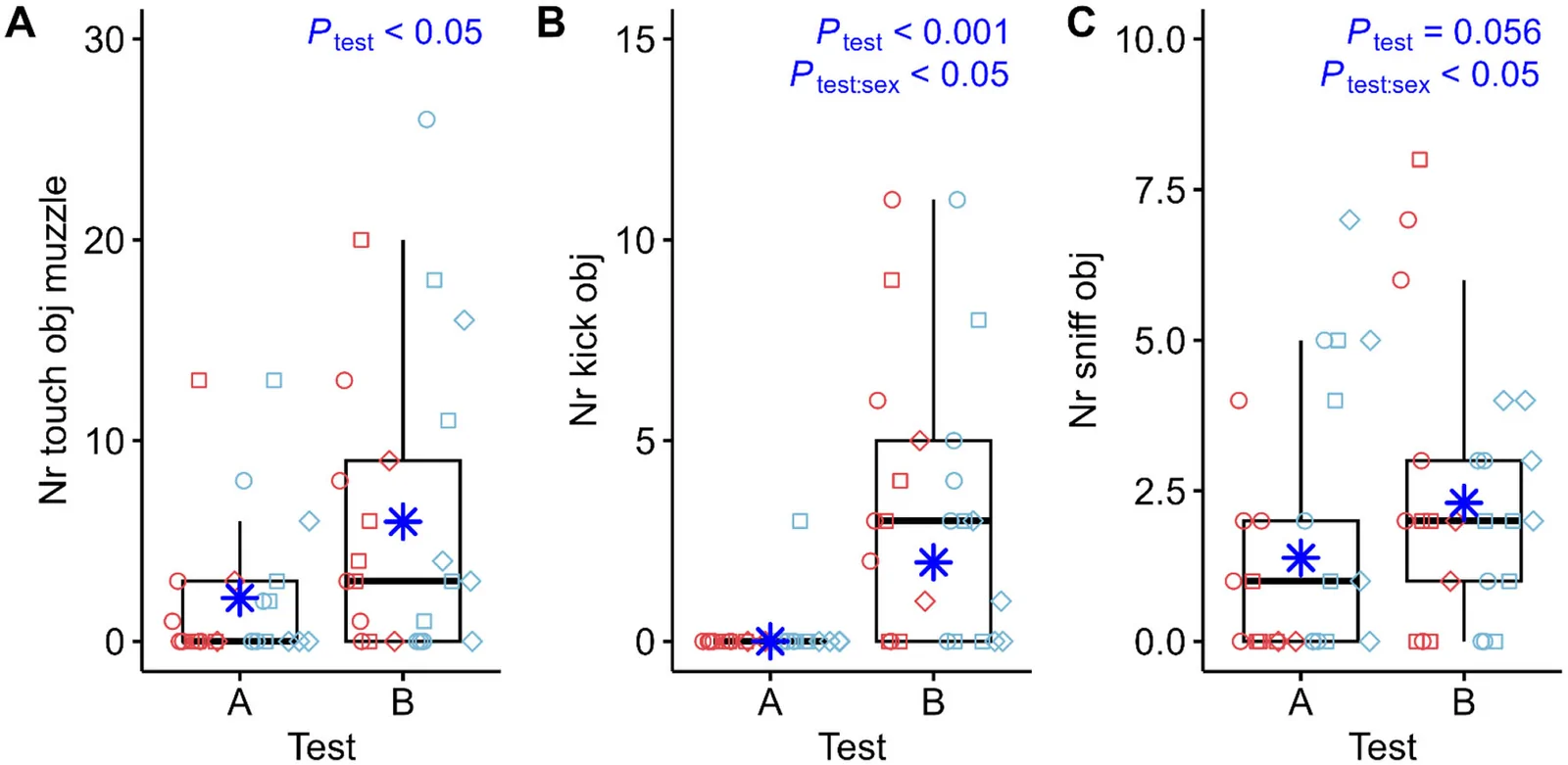
**(A)** Number of touching the object with the muzzle, **(B)** number of kicking toward the object, and **(C)** number of sniffing the object compared between Test A (first novel object test) and Test B (second novel object test) (*n* = 25). The box plots display the distribution of data by indicating the median and the interquartile range (IQR). Individual data points from mares (*n* = 12) are either shown as red circles (novel object test with object “fish”), red squares (novel object test with object “lama”) or red trapezoids (novel object test with object “blue”). Individual data points from stallions (*n* = 13) are shown in blue circles, squares and trapezoid accordingly. The marginal means estimated by the mixed effects models are shown as blue asterisks. *P*-values result from negative-binomial regression models.

### 3.2 Activity analysis

The results of the activity recordings from the horses' stalls during the days on which the novel object tests were conducted revealed relevant differences in the time budgets of certain behaviors. At the time of the second novel object test, the horses spent a greater amount of time eating from the trough (Figure 6A). An interaction between time point and sex was identified, with pairwise comparisons indicating that this difference was primarily observed in mares. Furthermore, the horses spent less time walking around in the box (Figure 6B) and standing alert (Figure 6C) at the time point of the second novel object test. A significant interaction between time point and sex revealed that stallions spent more time walking and in recumbency (Figure 6D).

**Figure 6:**
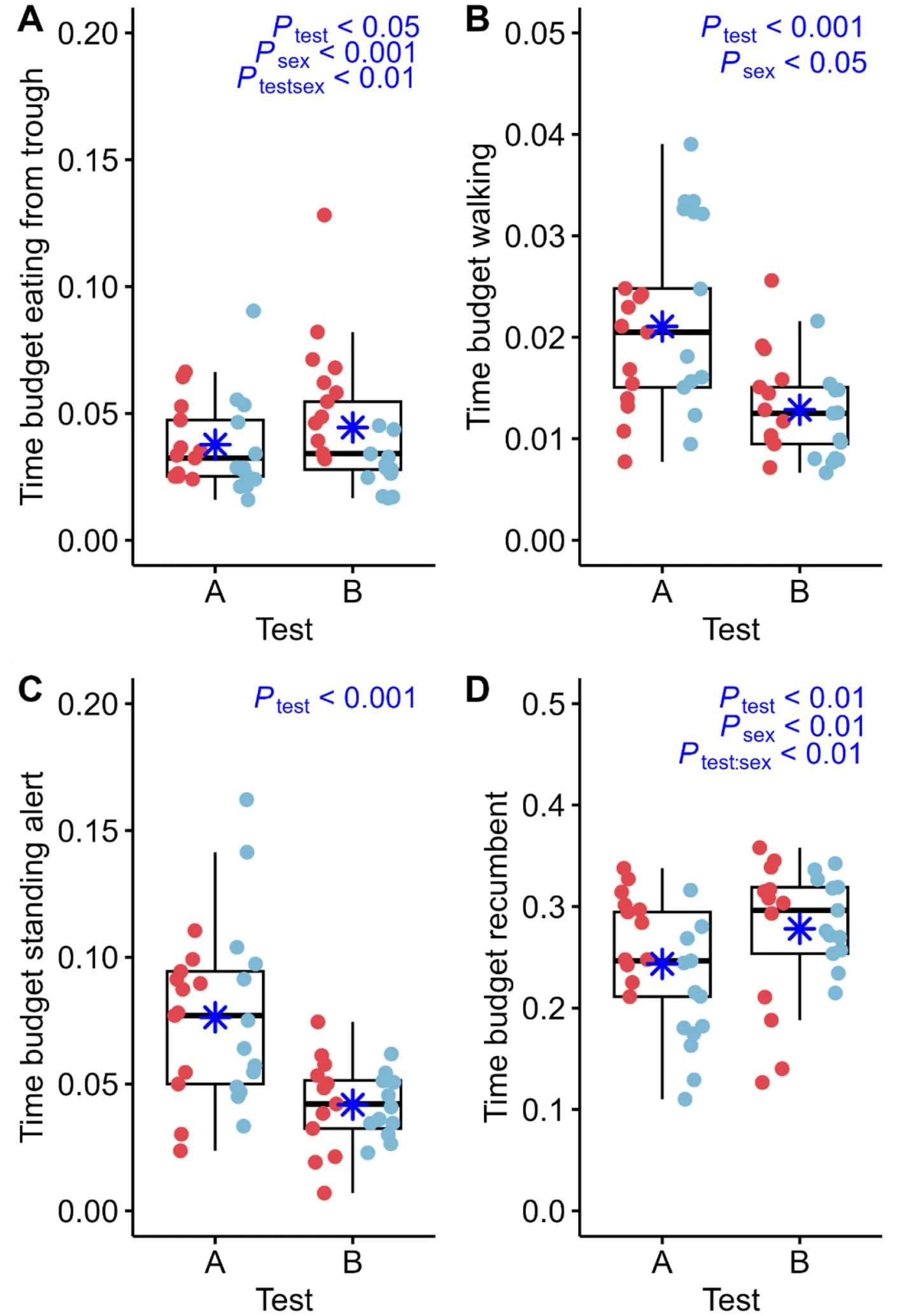
The time budgets of **(A)** eating from the trough, **(B)** walking, **(C)** standing alert, and **(D)** recumbency in the stall are shown as a proportion of an hour compared between Test A (first novel object test) and Test B (second novel object test) (*n* = 25). The box plots display the distribution of data by indicating the median and the interquartile range (IQR). Individual data points from mares (*n* = 12) are shown as red circles. Individual data points from stallions (*n* = 13) are shown in blue circles. The marginal means estimated by the mixed effects models are shown as blue asterisks. *P*-values result from negative-binomial regression models.

### 3.3 Principal component analysis of the ethogram and activity analysis

Cluster analysis was performed using the first 12 principal components accounting for 80% of the variation in the data. The resulting clusters are visualized in the loadings plot of the first two principal components (Figure 7A). The principal component and k means cluster analysis show that certain behavioral parameters that have been observed during the novel object test were more associated with each other than others and formed clusters of parameters, that jointly contribute to the principal components. In addition to the data from the novel object test, behavioral data from the box were also integrated into this analysis. Four main clusters of highly associated behaviors could be identified and are visualized in Figure 7A. The green cluster summarizes a group of 10 behaviors associated with high activity. These include trotting, galloping, bucking, head tossing and increased vocalization, such as squeaking. The behaviors also correlate with the duration of recumbency in the stable. The orange cluster summarizes 14 behaviors. This cluster includes behaviors associated with increased exploratory behavior, such as sniffing, approaching, and touching the object, as well as exploring the test environment by sniffing the ground, rolling, snorting, and shaking. It is also associated with the duration spent standing and sleeping in the stall, as well as duration spent eating from the ground in the stall. The purple cluster is associated with increased fixation on the object, standing alert in the box, and standing alert during the novel object test. Additionally, associations between walking in the box and during the novel object test are found in this cluster. Furthermore, this cluster includes the number of defecations and the latency time until the first touch of the object. The blue cluster is characterized by less active behaviors. Here, the horses wait at the exit and stand and neigh. Other associations in this cluster are latency to first approach the object and the duration eating from the trough. When comparing the individual data points between the first test at the start of habituation and the second test at the end of pre-training (Figure 7B) and sexes (Figure 7C), it becomes apparent that the point cloud for the second test shifted toward the right quadrants of the plot, with more active behavior and more bucking behavior. The cluster analysis revealed no significant sex-based distinctions. Furthermore, a higher inter-individual variability could be seen in the second novel object test.

**Figure 7:**
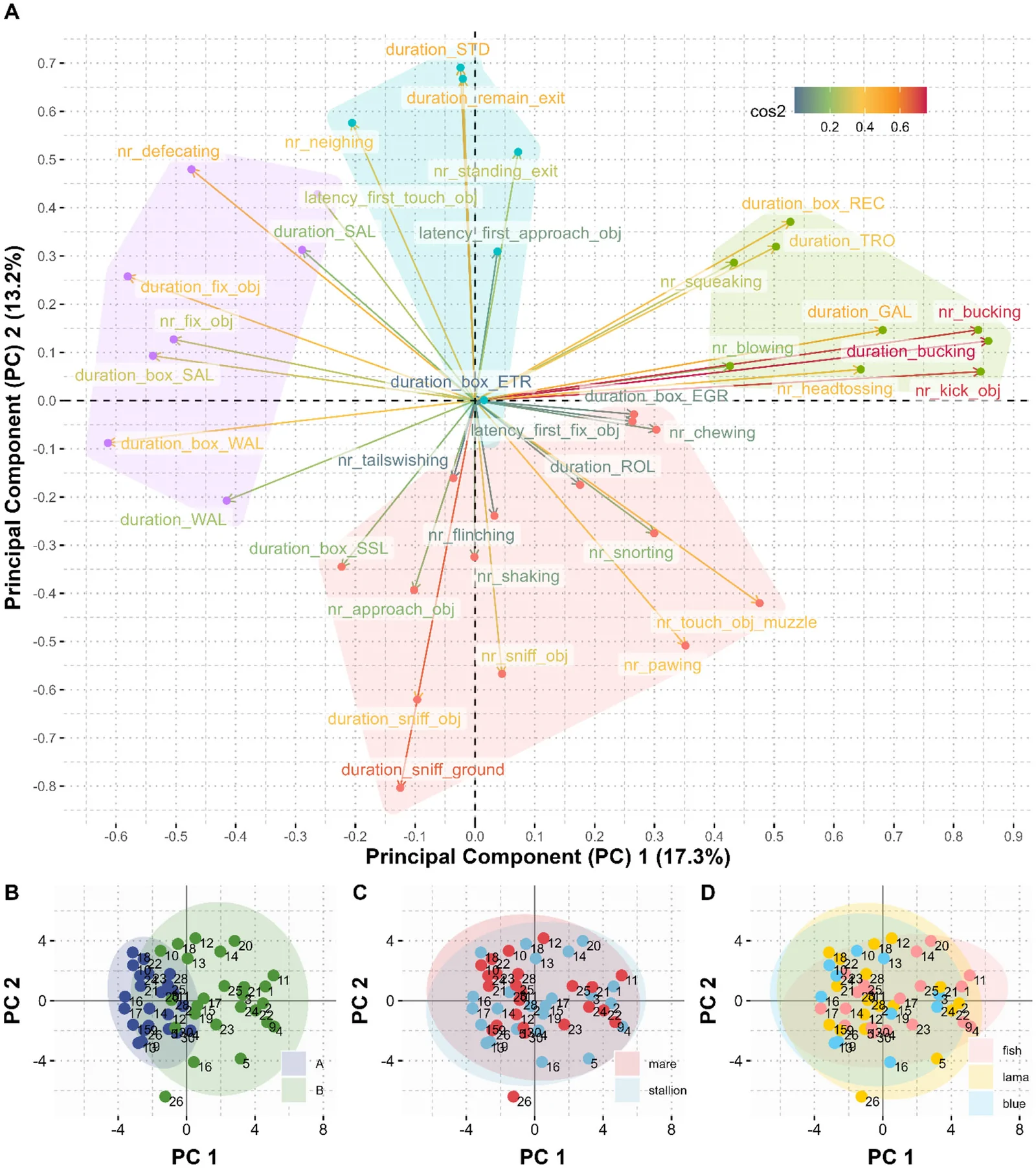
**(A)** Correlation (loadings) between the different behavioral variables in the box and novel object test and the first (PC 1) and second principal component (PC 2). The variation explained by the principal components is given in % of total variability in the axe's labels. The quality of representation of the variables by the principal components (cos2) is indicated by the color of the arrows and labels. The further away a variable is from the origin of the plot, the more important it is to interpret these components. The closer to the center of the plot a variable is, the less important it is for the first two components. Variables whose arrows point in the same direction are positively correlated and variables whose arrows point in opposite directions are negatively correlated to each other. The clusters identified by means of k-means clustering of the coordinates of the variables on the first 12 principal components (eigenvalue >1) are indicated by different coloring of the data points associated with the variables. Individual data points for PC 1 and PC 2 compared between **(B)** the first novel object tests (dark blue) or second novel object test (green), and compared between **(C)** mares (red) and stallions (light blue), and compared between **(D)** the objects fish (red), lama (yellow) and blue (blue).

## 4 Discussion

The results of this study reveal clear behavioral differences between the first and second novel object tests. After the pre-training phase, horses displayed increased activity, which was reflected in more frequent trotting and galloping as well as a marked rise in bucking behavior. Increased locomotion and heightened vigilance are behavior patterns that are typically associated with heightened arousal or fearfulness and have previously been linked to stress-related responses (13, 14). Since the horses were tested individually, social separation-a known stressor in equines (15)-may have amplified this behavioral pattern. The principal component analysis corroborates these findings, showing a significant difference in PC1, which reflects elevated activity. One plausible explanation for this increase may be the need to dissipate accumulated energy, especially in young horses kept individually. Stored energy resulting from such housing conditions has been associated with increased bucking behavior (16). The observation that horses spend a greater proportion of time-consuming feed from the trough toward the end of the pre-training phase may indicate a greater intake of concentrated feed, potentially further contributing to higher energy levels and, consequently, increased activity during the second test.

A rise in defensive behavioral elements, such as kicking toward the novel object, has been observed. Kicking with the hindlegs is considered part of equine threat behavior (17), and may indicate that the unfamiliar object was perceived as a potential threat. These behaviors may, however, not solely be interpreted as defensive responses. They can also be attributed to conflict behavior, which represents a form of displacement behavior occurring when a strong motivation cannot be discharged (18). Such conflict behavior emerges, for example, when the housing system restricts the expression of innate behavioral needs, or when training and handling repeatedly overwhelm the horse (18). In this context, the increased kicking and bucking observed here may reflect a mixture of defensive arousal and motivational conflict, potentially indicating compromised wellbeing. Notably, however, no increase in defecation was observed. While defecation can occur in both positive and negative arousal states (19) it may also serve as a form of intraspecific communication (18). Its reduction in the second test could therefore reflect lower excitement. Suggesting that the arousal state did not manifest uniformly across all fear- or stress-related behaviors. For this reason, it is important to pay special attention to the object-relating behaviors.

In contrast to PC1, no significant difference was found for PC2, which represents exploratory behavior. Although the horses showed longer latencies to first fixation and reduced duration and frequency of visual fixation in the second test, they simultaneously touched the object more frequently with their muzzles. Limited interaction with a novel object is typically interpreted as a fear response (9), whereas curious horses display more object-directed exploratory behavior (13). Direct touching-especially sniffing or nibbling-has been regarded as an indicator of reduced fear (20). Thus, the pattern observed here, namely fewer approaches but more tactile interactions, may point to reduced anxiousness during actual engagement with the object, even if initial hesitancy remained.

Exploration of the test site itself decreased markedly in the second test, as indicated by the reduced sniffing behavior directed at the ground. This suggests that the horses were less focused on investigating the surroundings, possibly because the overall testing environment was no longer entirely unfamiliar (21).

It may be hypothesized that the pre-training could have functioned as a form of enrichment by offering cognitive stimulation, predictable human–animal interactions, and controlled physical activity, thereby potentially promoting exploratory motivation. The increased willingness to touch the object, despite fewer overall approaches, may therefore indicate enhanced confidence or curiosity arising from the training experience. However, distinguishing the effects of pre-training from the influence of housing conditions remains challenging. The young horses in this study were kept in individual boxes, a system that does not fully meet their ethological needs and is considered unsuitable for horses of this age according to current husbandry guidelines (22), which recommend group housing for young equines. Although access to pasture with conspecifics provided partial compensation for the lack of continuous social contact, it cannot fully replace the behavioral and social benefits of permanent group housing. Consequently, some of the observed behaviors-such as increased activity, bucking or elements of conflict and displacement behavior-may reflect a combination of pre-training-related effects and the constraints of individual housing.

The present results go along with the findings from Christensen et al. (8) suggesting that habituation is highly object-specific and that a considerable degree of similarity between objects is required for generalization to occur. In this study, the novel objects used in the second novel object test differed from the first test object (mermaids tail) as follows: the object “blue” shared the same color, the object “lama” a similar shape, while the object “fish” differed in both color and shape. Across all objects, prolonged bucking duration and higher frequency of bucking behavior was observed in the second test; however, the frequency of bucking increased more in the presence of the “fish” compared to the other objects as indicated by the significant interaction between test and object. In the present study, the object that differed most clearly from the original object (“fish”) appeared to elicit the highest level of arousal, whereas the object with identical color (“blue”) triggered the lowest reaction. The shape-similar object (“lama”) elicited an intermediate response. These patterns support the assumption that equine arousal is influenced by the degree of novelty or deviation in object features. The shape and color of the objects used in novel object tests should therefore be considered as potential influencing factors. However, general differences in the behavioral responses between the first and the second test could be observed independently of the object.

The sensor data collected in the stalls provides additional insights into the horses' behavioral state on the days of the novel object test. On the day before the beginning of the pre-training phase, the horses exhibited heightened vigilance and increased movement, which manifested as frequent walking within the stall. Following the completion of the pre-training phase, however, the horses appeared noticeably calmer in their stalls. One possible interpretation of these findings is that the animals initially required time to acclimatize to the unfamiliar environment. Alternatively, the observed reduction in stall activity may reflect a beneficial influence of the pre-training, which could have contributed to a more stable emotional state. Throughout the study period, all horses spent an appropriate amount of time lying down, with stallions showing a particularly notable increase in recumbent behavior. Since horses achieve REM sleep only in a recumbent position, recumbency serves as an important welfare indicator (23, 24), these findings may indicate that the animals felt sufficiently safe and adapted to the housing conditions as the study progressed. This interpretation is supported by Greening and McBride (25), who argue that external stressors and environmental changes can influence both the duration and the quality of equine sleep.

The cluster analysis provides additional insight into how individual behavioral parameters relate to one another across both the novel object test and stall observations. The identified clusters indicate that certain behaviors consistently co-occur. The green cluster, representing highly active behavior, comprises various locomotor and arousal-related responses. Its association with increased recumbency in the stall may appear counterintuitive at first. However, it may possibly reflect alternating periods of heightened arousal and subsequent compensatory recovery phases. Horses from this cluster could broadly be described as active. The orange cluster summarizes behaviors associated with exploratory motivation. It includes object-directed exploration (approaching, sniffing and touching) and investigation of the test environment. Its association with standing and sleeping in the stall as well as eating from the ground may indicate that this cluster reflects a behavioral profile characterized by calmness, curiosity and environmental engagement. The purple cluster is composed of behaviors linked to vigilance and focused attention. Increased fixation on the object, standing alert in both the stall and the novel object test arena, and walking behavior are all part of this group. Horses seem to be more defensive in this cluster. The inclusion of defecation frequency and latency to first touch of the novel object suggests that this cluster represents a state of heightened attentiveness and moderate arousal, potentially associated with uncertainty or ambivalence toward the novel stimulus. Horses in this cluster may be monitoring their environment closely while hesitating to approach the object directly. Cluster blue encompasses individuals showing calm and observant behavior patterns. This cluster is characterized by behaviors associated with low activity levels, including waiting at the exit, standing and neighing. The presence of latencies to first approach and first touch within this cluster supports the interpretation that these behaviors reflect avoidance tendencies or low motivation to interact with the object. The analysis further suggests that some horses that showed more defensive tendencies in the first test appeared to shift toward more active behavior in the second test. This behavioral change may potentially reflect the enriching effects of the pre-training phase, which could have increased the horses' confidence or reduced their sensitivity to novelty. However, other behavioral tendencies appeared more stable: horses assigned to the calm/observant cluster or the exploratory cluster showed no evidence of shifting between these profiles. This pattern supports the notion that certain behavioral strategies may be more trait-like, whereas others may be more susceptible to modification through experience or training-related enrichment.

Notably, the cluster analysis did not reveal significant sex-related differences, supporting earlier observations that sex does not influence direct object interaction (6). However, the greater inter-individual variability observed in the second novel object test indicates that horses responded more variably after the pre-training phase, which may reflect differences in individual coping strategies, prior experience, or sensitivity to the training process. Overall, the cluster analysis highlights the multifaceted nature of equine responses to novelty. Rather than functioning as isolated indicators, behavioral parameters combine into coherent profiles that offer promising opportunities for deeper insights into emotional states, arousal dynamics and individual coping styles in horses.

The present study has several limitations that should be considered when interpreting the results. A repeated-measures design was used, comparing behavioral responses before and after an approximately 4-month pre-training period within the same individuals; however, the absence of a non-trained control group limits causal inference, as the observed differences may also reflect natural maturation, adaptation to the housing environment, habituation to the experimental procedure, or the accumulation of experience over time rather than the effects of pre-training alone. To reduce potential habituation effects, different, previously unseen novel objects were used during the second novel object test, and the interval between assessments was at least 12 weeks. Nevertheless, a contribution of habituation to the observed behavioral changes cannot be completely excluded. In addition, the inclusion of a larger cohort would have been desirable to increase statistical power and improve the generalizability of the findings, particularly given the inherent inter-individual variability in equine behavior. Although the inclusion of a larger sample size and a non-trained control group would have strengthened the experimental design, this was not feasible for practical and ethical reasons. In accordance with the 3R principles, animal numbers were kept to a minimum, and establishing a control group would have required additional horses, as maintaining Thoroughbreds in a pre-training environment without training would have resulted in considerable financial disadvantages for their owners.

## 5 Conclusion

Overall, the results demonstrate that horses exhibited altered behavioral responses following the pre-training phase. In the novel object test, horses showed increased locomotor activity and more tactile interactions with the novel object, indicating changes in their responses to novelty. Principal component and cluster analyses identified four distinct behavioral profiles and revealed increased inter-individual variability after pre-training, suggesting that horses differed in their behavioral adaptation to this phase. Furthermore, correlations between stall behavior and novel object test responses indicate that individual behavioral tendencies are expressed consistently across different contexts.

These findings highlight the value of evaluating multiple behavioral parameters simultaneously rather than relying on single measures when interpreting responses in the novel object test. Future studies should investigate the long-term stability of the identified behavioral profiles and their relationship with welfare and training outcomes.

## Data Availability

The raw data supporting the conclusions of this article will be made available by the authors upon request.
